# Longitudinal analysis of the patient pathways to diagnosis of psoriatic arthritis

**DOI:** 10.1186/s13075-021-02628-2

**Published:** 2021-10-01

**Authors:** Alexis Ogdie, Martin Rozycki, Theresa Arndt, Cheng Shi, Nina Kim, Peter Hur

**Affiliations:** 1 Perelman School of Medicine at the University of Pennsylvania, Philadelphia, PA USA; 2 HVH Precision Analytics LLC, Wayne, PA USA; 3 Novartis Pharmaceuticals Corporation, East Hanover, NJ USA; 4 The University of Texas at Austin, Austin, TX USA; 5 Baylor Scott and White Health, Temple, TX USA

## Abstract

**Background:**

The occurrence of health events preceding a psoriatic arthritis (PsA) diagnosis may serve as predictors of diagnosis. We sought to assess patients’ real-world experiences in obtaining a PsA diagnosis.

**Methods:**

This retrospective cohort study analyzed MarketScan claims data from January 2006 to April 2019. Included were adult patients with ≥ 2 PsA diagnoses (ICD-9-CM/ICD-10-CM) ≥ 30 days apart with ≥ 6 years of continuous enrolment before PsA diagnosis. Controls were matched 2:1 to patients with PsA. Health events (diagnoses and provider types) were analyzed before PsA diagnosis and additionally stratified by presence of psoriasis.

**Results:**

Of 13,661 patients, those with PsA had an increased history of coding for arthritis and dermatologic issues (osteoarthritis [48% vs 22%], rheumatoid arthritis [18% vs 2%], and psoriasis [61% vs 2%]) vs those without PsA. Diagnoses of arthritis, axial symptoms, and tendonitis/enthesitis increased over time preceding PsA diagnosis; notably, a sharp rise in psoriasis diagnoses was observed 6 months before PsA diagnosis. Rheumatology consults were more common immediately preceding a PsA diagnosis. Dermatologists were unlikely to code for arthritis and musculoskeletal issues, while rheumatologists were unlikely to code for psoriasis; general practitioners focused on axial and musculoskeletal symptoms. PsA was most commonly diagnosed by rheumatologists (40%), general practitioners (22%), and dermatologists (7%).

**Conclusions:**

Rheumatologists, general practitioners, and dermatologists diagnosed two thirds of patients with PsA. Musculoskeletal symptoms were common preceding a PsA diagnosis. Greater awareness of patterns of health events may alert healthcare providers to suspect a diagnosis of PsA.

## Background

Psoriatic arthritis (PsA) is a chronic inflammatory disease affecting the skin and musculoskeletal system with various manifestations [1, 2]. PsA is typically characterized by 6 clinical domains: axial disorders, nail disease, skin disease, peripheral disease, enthesitis, and dactylitis, either alone or in combination [3]. PsA is also associated with many comorbidities [4] that collectively impair patients’ quality of life, overall function, and clinical burden [5]. A timely diagnosis of PsA is critical because a delay in diagnosis negatively impacts patient outcomes [6, 7].

Unfortunately, diagnostic delay is common in PsA. In a cross-sectional study of 203 participants with self-reported PsA in the USA, approximately one third received their diagnosis within 6 months to 4 years, while another one third had to wait for > 5 years [8]. These participants also consulted with numerous healthcare providers prior to receiving their diagnosis. A 6-month delay from PsA symptom onset to initial rheumatology consult may lead to joint erosion and damage [9, 10]. An increased awareness of the heterogeneity of PsA symptoms and diagnostic barriers may lead to a timely diagnosis.

Our study aimed to assess patient experiences in the period prior to diagnosis of PsA using administrative claims data. We described and analyzed health events (i.e., diagnosis codes and provider types consulted by patients) prior to PsA diagnosis to better understand a patient’s journey to diagnosis.

## Materials and methods

### Data source

This retrospective cohort study used administrative claims data from patients in the Truven MarketScan research databases [11] from January 2006 to April 2019. Comprising 3 core claims databases (Commercial, Medicare Supplemental, and Medicaid), the MarketScan records represent one of the largest collections of deidentified US patient data available for healthcare research, with > 263 million covered lives in 2020. Data sets include the inpatient, outpatient, and outpatient prescription drug experience of several million employees and their dependents (annually), covered under a variety of fee-for-service and capitated health plans, including exclusive provider organizations, preferred provider organizations, point-of-service plans, indemnity plans, and health maintenance organizations. These databases provide detailed cost, utilization, and outcomes data for healthcare services performed in both inpatient and outpatient settings. The medical claims are linked to outpatient prescription drug claims and person-level enrolment data using unique enrollee identifiers.

### Data variables and study population

Variables considered in this study included health events and demographics extracted entirely from the claims database; no prefiltering of any variable category was performed. To be included in our study, patients with PsA must have received 2 diagnoses of PsA, specified by the International Classification of Diseases, Ninth and Tenth Revisions, Clinical Modification (ICD-9-CM and ICD-10-CM), ≥ 30 days apart to account for potential clustering of care immediately following the first diagnosis of PsA and increase the confidence that the diagnosis is correct [12–14]; patients were aged ≥ 18 years at diagnosis and had ≥ 6 years of continuous enrolment prior to PsA diagnosis. The following diagnosis codes were included: 696.0 (psoriatic arthropathy), L40.50 (arthropathic psoriasis, unspecified), L40.51 (distal interphalangeal psoriatic arthropathy), L40.52 (psoriatic arthritis mutilans), L40.53 (psoriatic spondylitis), L40.54 (psoriatic juvenile arthropathy), and L40.59 (other psoriatic arthropathy). Controls (those without a diagnosis of PsA) were matched 2:1 to patients with PsA for age, sex, insurance, and enrolment duration (i.e., non-PsA controls were enrolled in the database for the same length of time as their associated PsA patient so that the index date for controls was the end of the matched ≥ 6-year period that terminated in “no PsA”).

The data analyzed in this study included the demographic variables mentioned above as well as data on health events. A health event was defined as any health service provided to a patient in the preindex period, derived from their medical and pharmacy claims history. Health events included PsA diagnosis codes (ICD-9-CM and ICD-10-CM) and specialty physician types (e.g., general practitioners, dermatologists, rheumatologists) as enumerated by the MarketScan databases. Information on health events was captured every 6 months for the 6 years prior to PsA diagnosis. Results were also stratified by the presence of psoriasis vs no psoriasis prior to PsA diagnosis.

This study was conducted in accordance with the Guidelines for Good Pharmacoepidemiology Practices of the International Society for Pharmacoepidemiology, the Strengthening the Reporting of Observational Studies in Epidemiology guidelines, and the ethical principles in the Declaration of Helsinki. All database records were fully compliant with US patient confidentiality requirements, including the Health Insurance Portability and Accountability Act of 1996. As this study is a retrospective analysis of deidentified claims data in compliance with US patient confidentiality requirements and did not involve the collection, use, or transmittal of individually identifiable data, institutional review board approval was not required to conduct this study.

## Results

### Patient demographics and diagnosis history

Of > 265 million patients in the MarketScan databases, 280,311 patients had ≥ 1 diagnosis of PsA and 174,524 patients had ≥ 2 diagnoses of PsA 30 days apart, of whom 172,119 patients were aged ≥ 18 years at the index date. Of these, a final cohort of 13,661 patients had ≥ 6 years of continuous enrolment in the database, fulfilling the criteria for inclusion in the study. The mean age was 55.8 years and 60.2% were women; baseline demographics of patients diagnosed with PsA and non-PsA controls are shown in Table 1.

**Table 1:** Baseline demographics of patients diagnosed with PsA and non-PsA controls

Characteristic	Patients with PsA (***N*** = 13,661)	Non-PsA controls^**a**^ (***N*** = 26,729)
Age, mean (SD), years	55.8 (12.6)	55.9 (12.7)
Female, %	60.2	60.4
Insurance type, %		
Commercial	78.6	78.6
Medicaid	15.1	15.3
Medicare	6.3	6.1
US geographical region, %		
Midwest	21.7	30.0
Northeast	15.2	48.7
South	43.6	11.9
West	12.9	3.2
Unknown	6.6	6.2

*PsA* Psoriatic arthritis

^a^Controls were matched 2:1 to patients with PsA for age, sex, insurance, and enrolment duration

**Fig. 1:**
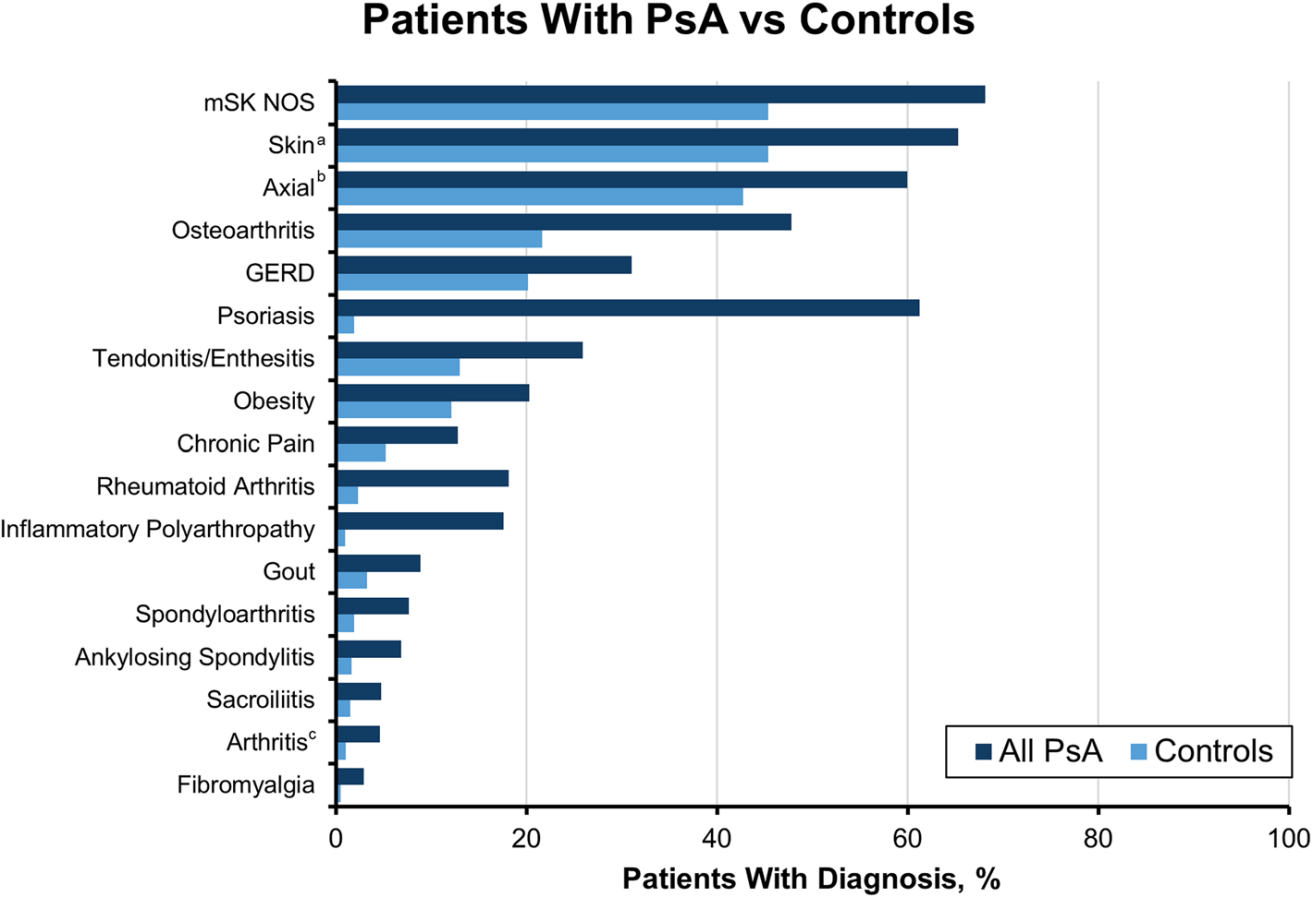
Diagnosis codes in the 6 years prior to PsA diagnosis among patients with PsA vs controls. GERD, gastroesophageal reflux disease; mSK, musculoskeletal; NOS, not otherwise specified; PsA, psoriatic arthritis. ^a^Skin comprises 43 codes, including dermatitis, rash, acne, and keratosis. ^b^Axial codes include > 50 codes relating to axial disorders and syndromes and includes codes for spondylopathy, backache, and disc disorders. ^c^Arthritis codes include chronic postrheumatic arthropathy (714.4); synovitis and tenosynovitis, unspecified (727.00); synovitis and tenosynovitis in diseases classified elsewhere (727.01); and secondary multiple arthritis (M153)

**Fig. 2:**
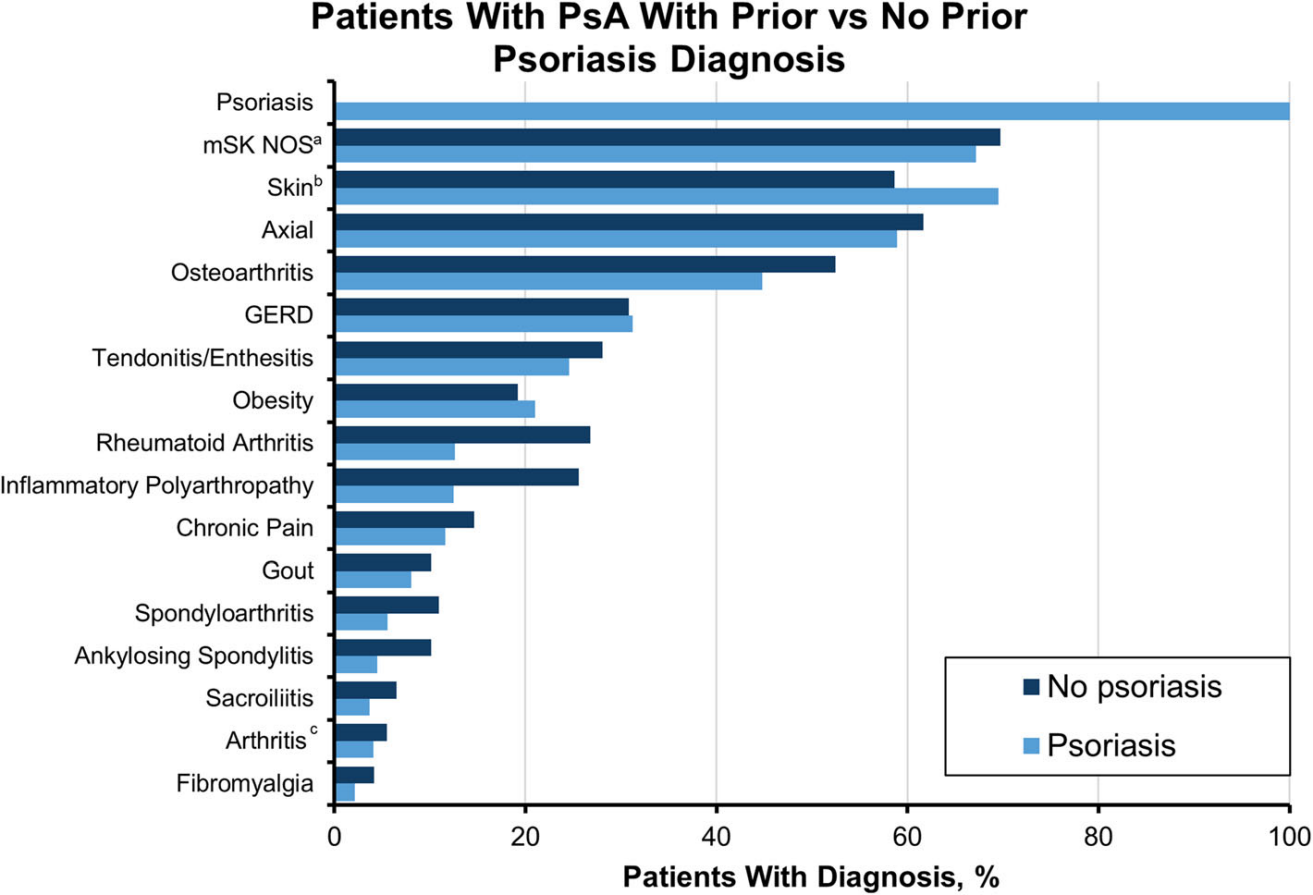
Diagnosis codes in the 6 years prior to PsA diagnosis among patients with PsA with prior vs no prior psoriasis diagnosis. GERD, gastroesophageal reflux disease; mSK, musculoskeletal; NOS, not otherwise specified; PsA, psoriatic arthritis. ^a^Skin comprises 43 codes, including dermatitis, rash, acne, and keratosis. ^b^Axial codes include > 50 codes relating to axial disorders and syndromes and includes codes for spondylopathy, backache, and disc disorders. ^c^Arthritis codes include chronic postrheumatic arthropathy (714.4); synovitis and tenosynovitis, unspecified (727.00); synovitis and tenosynovitis in diseases classified elsewhere (727.01); and secondary multiple arthritis (M153)

Compared with controls, patients with PsA received more codes for arthritis and skin issues, including osteoarthritis (OA; 47.8% vs 21.7%), rheumatoid arthritis (18.1% vs 2.3%), inflammatory polyarthropathy (IA; 17.6% vs 1.0%), and psoriasis (61.3% vs 1.9%) (Fig. 1). Patients with PsA without a prior diagnosis of psoriasis received more codes for other forms of arthritis compared with those with PsA and psoriasis, including OA (52.5% vs 44.8%), rheumatoid arthritis (26.8% vs 12.6%), and IA (25.6% vs 12.5%) (Fig. 2).

**Fig. 3:**
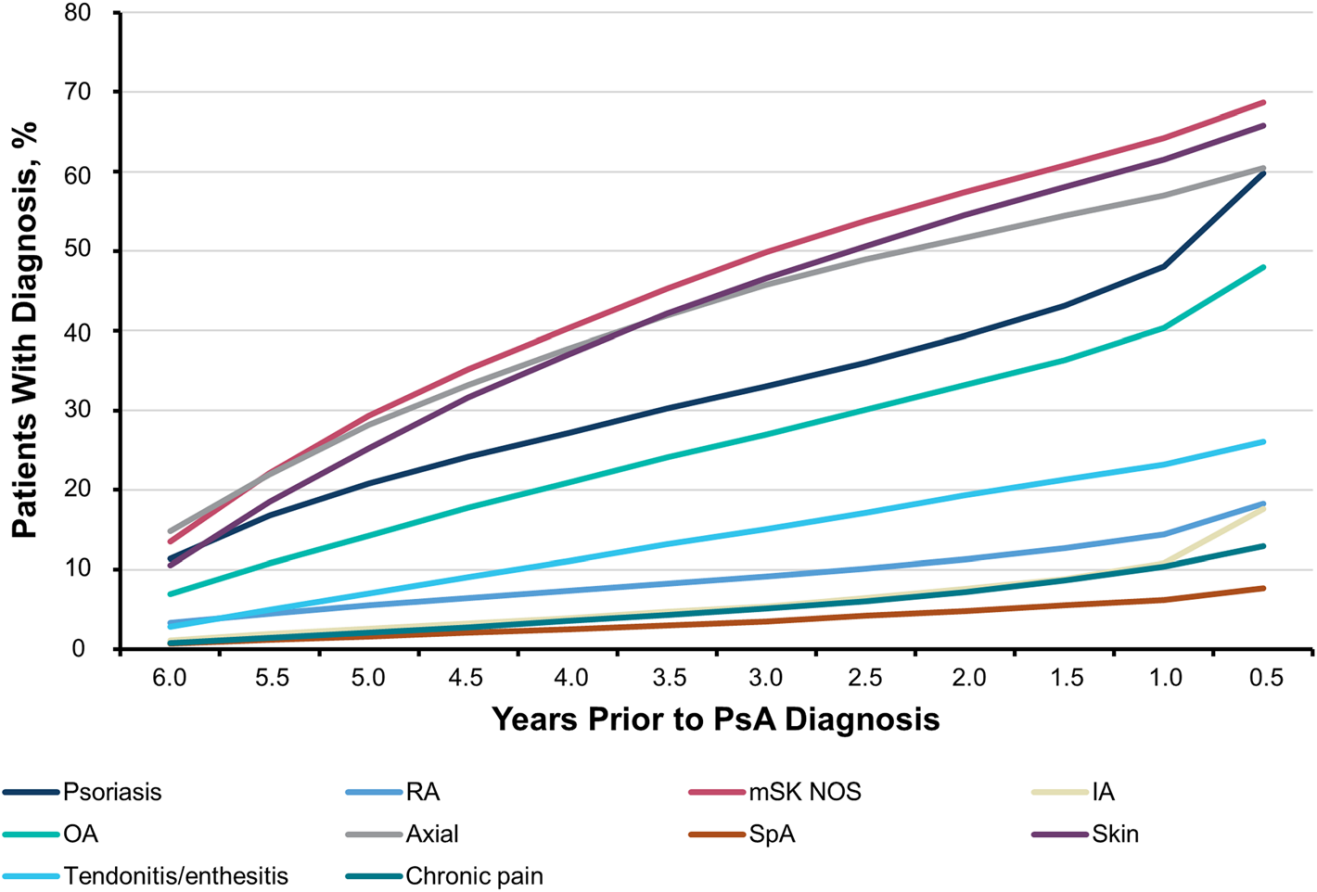
Cumulative patient journey through different diagnoses in the 6 years prior to PsA diagnosis. IA, inflammatory polyarthropathy; mSK, musculoskeletal; NOS, not otherwise specified; OA, osteoarthritis; PsA, psoriatic arthritis; RA, rheumatoid arthritis; SpA, spondyloarthritis

Patients received a variety of diagnosis codes for their condition prior to the first PsA diagnosis code, suggestive of misdiagnosis. These patients received many symptom codes over the 6-year period prior to PsA diagnosis (Fig. 3). More than 10% of patients had coding for axial manifestations, nonspecific musculoskeletal manifestations, psoriasis, and/or skin manifestations 6 years prior to receiving their PsA diagnosis; by the time patients were diagnosed with PsA, > 20% of patients had diagnoses of nonspecific musculoskeletal manifestations, skin manifestations, axial manifestations, psoriasis, OA, and/or tendonitis/enthesitis. In the final 6-month period prior to PsA diagnosis, a sharp rise in the cumulative number of psoriasis diagnoses was observed (48.0 to 60.5%); larger increases in the number of diagnoses of OA (40.4 to 48.0%) and IA (10.8 to 17.6%) were also noted in the same timeframe.

### Physician specialty analysis

**Fig. 4:**
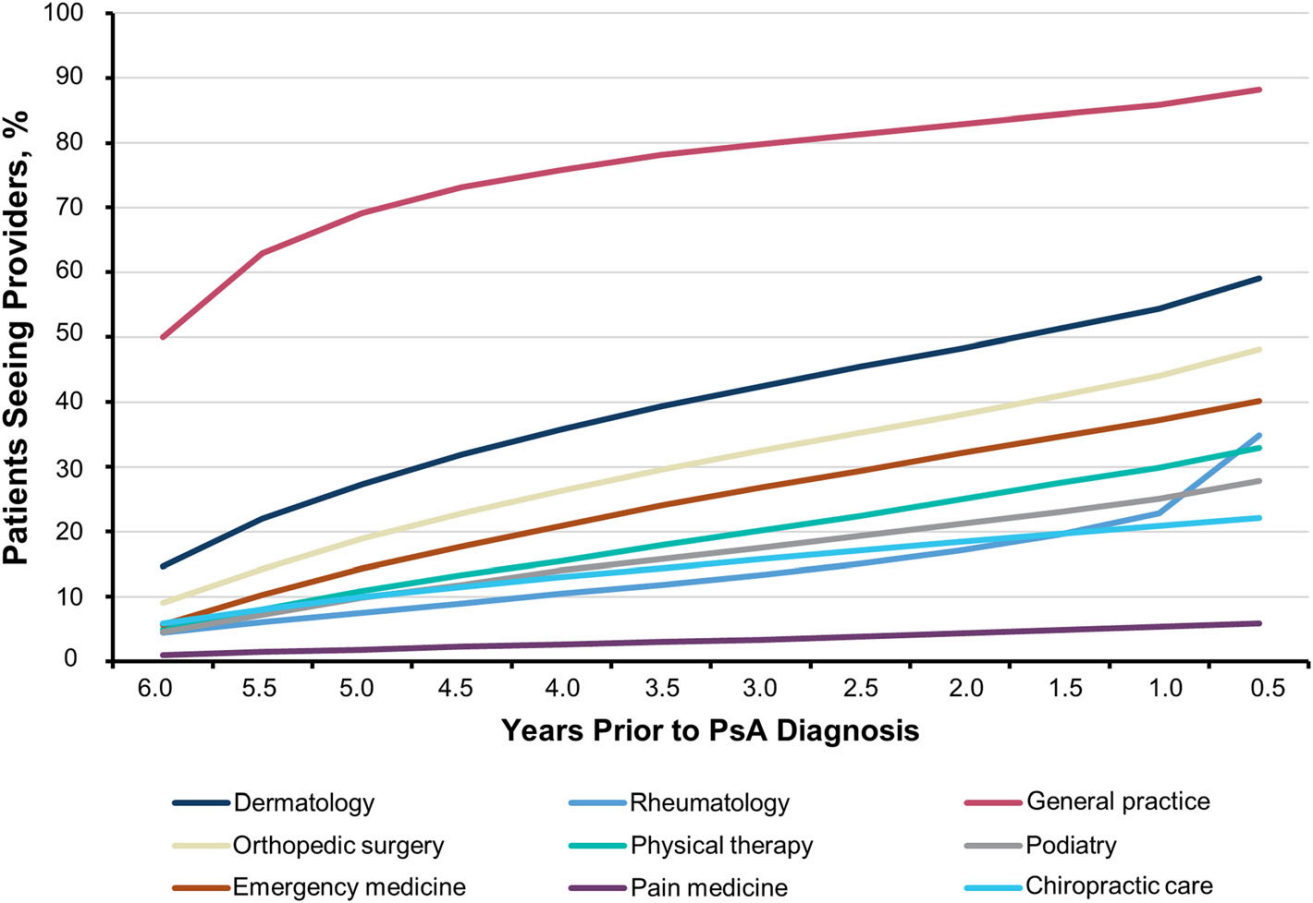
Cumulative patient journey through different providers in the 6 years prior to PsA diagnosis. PsA, psoriatic arthritis

General practitioners, rheumatologists, and dermatologists were seen with increasing frequency approaching diagnosis (Fig. 4). A large proportion of patients received medical care from general practitioners in the 6 years preceding the PsA diagnosis, with the cumulative proportion of patients increasing from 50.0% at 6 years prior to PsA diagnosis to 88.1% at 6 months prior to diagnosis. In this 6-year window, patients were symptomatic and received various non-PsA diagnosis codes for their condition, as shown in Fig. 3. A sizable and abrupt increase in the number of rheumatology visits was observed in the final 6-month period immediately before the diagnosis of PsA (22.9 to 34.9%). Large increases were also observed for dermatologist (54.4 to 59.0%) and orthopedic surgeon (44.1 to 48.2%) visits over the same time period.

**Fig. 5:**
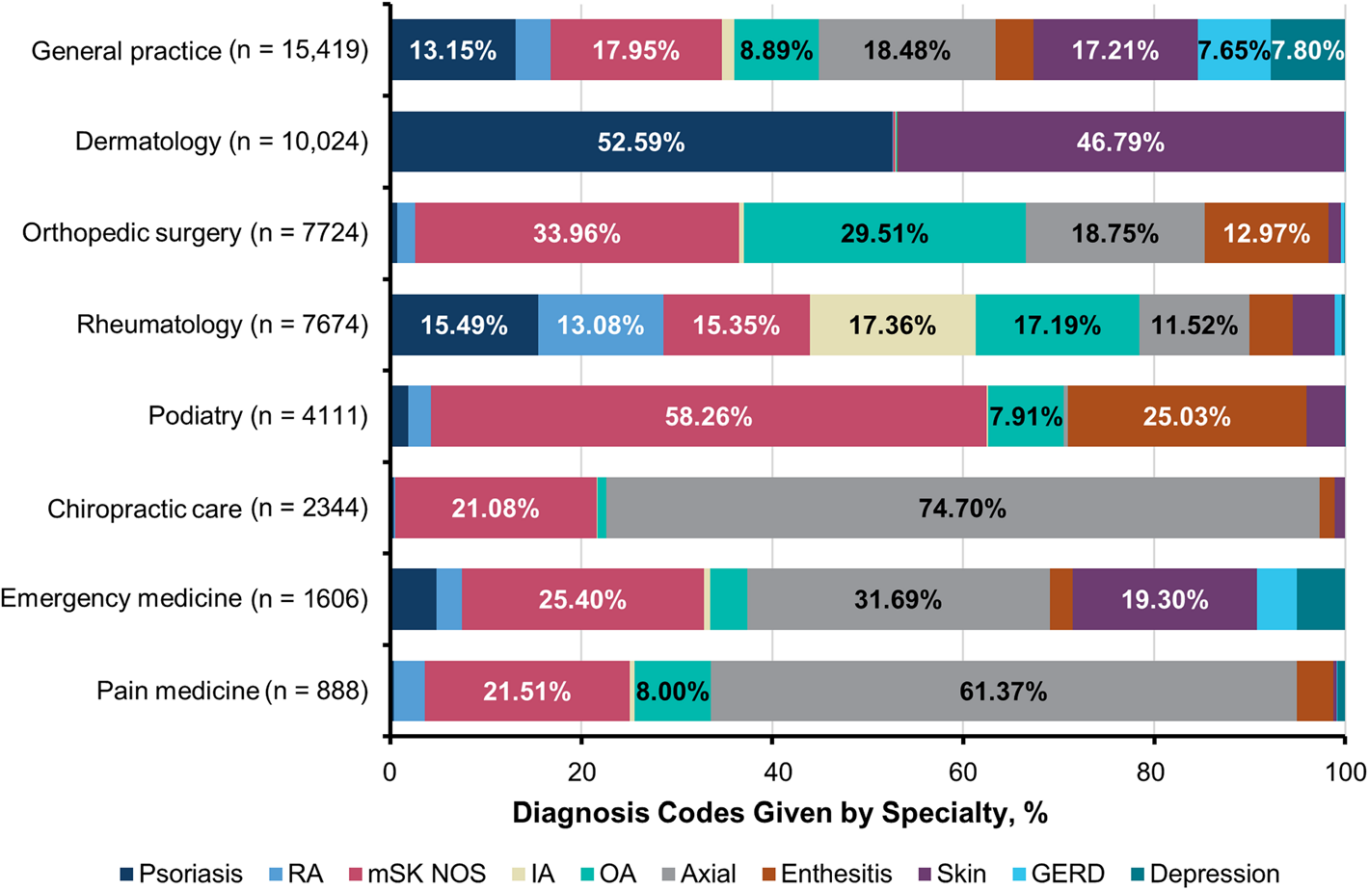
Coding patterns by specialty—fractions by specialty in the 6 years prior to PsA diagnosis. GERD, gastroesophageal reflux disease; IA, inflammatory polyarthropathy; mSK, musculoskeletal; NOS, not otherwise specified; OA, osteoarthritis; PsA, psoriatic arthritis; RA, rheumatoid arthritis

**Fig. 6:**
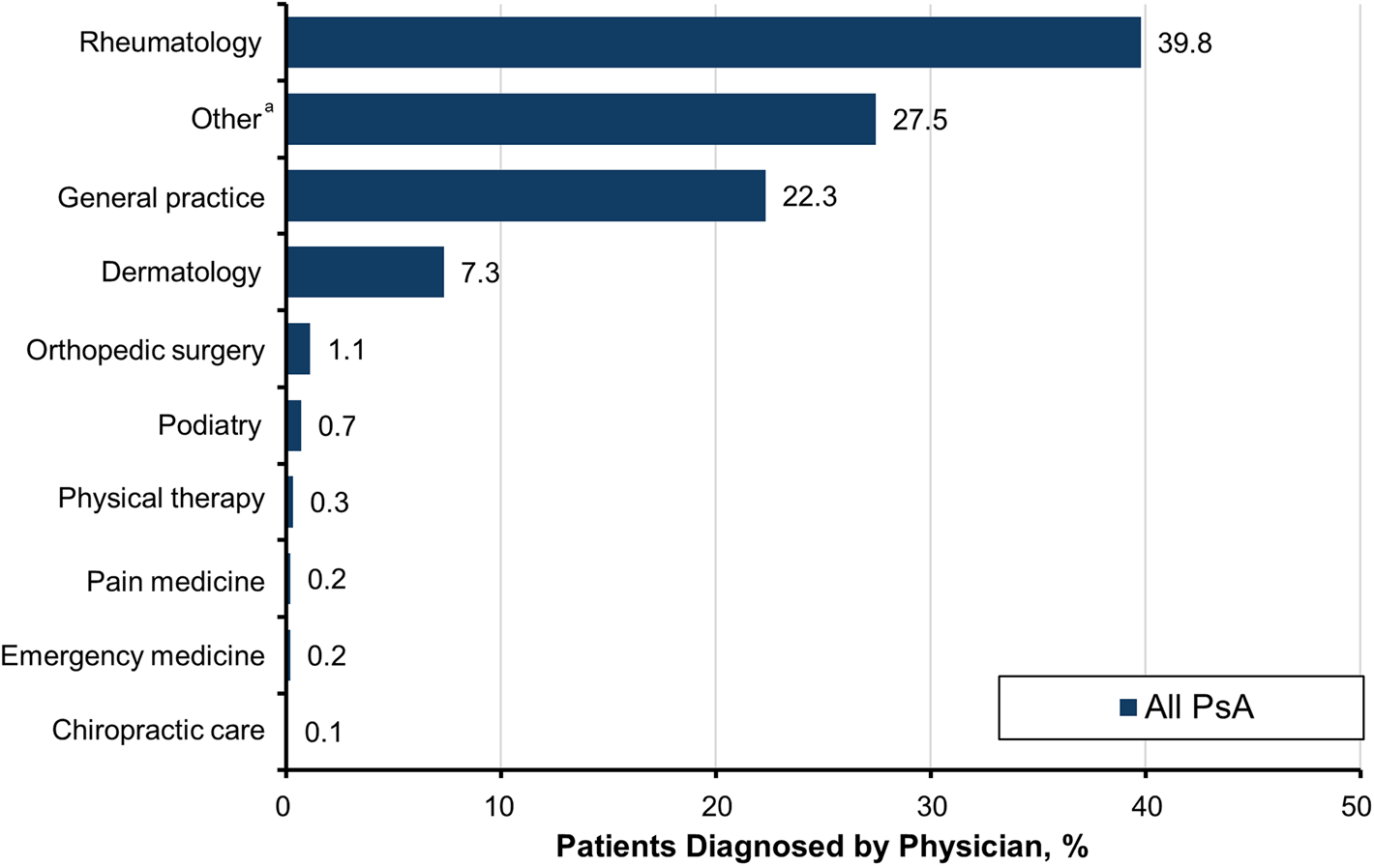
Distribution of physicians who diagnosed patients with PsA. PsA, psoriatic arthritis. ^a^Other comprises 71 provider types from other disciplines, including pathology, radiology, endocrinology, and ophthalmology based on provider codes, with most provider types contributing a very small percentage of the patients diagnosed. The top 4 provider types in the “other” category were “laboratory” (11.0%), “medical doctor/MD” (4.9%), “multispecialty physician group” (3.9%), and “other facility” (3.0%). These contribute to the “other” group without providing specific information on the type of provider making the diagnosis and thus were grouped as “other” to focus on those physicians who routinely encounter patients with PsA

Diagnoses made prior to the diagnosis of PsA differed by the type of provider that patients saw (Fig. 5). Dermatologists were less likely than other providers to enter codes for arthritis and musculoskeletal issues (< 1%), while rheumatologists were unlikely to code for psoriasis (15.5%) but had a fairly even distribution across different types of arthritis (IA, 17.4%; OA, 17.2%; and RA, 13.4%). General practitioners focused more on axial symptoms (18.5%) and nonspecific musculoskeletal manifestations (18.0%) than arthritis diagnoses. PsA was most commonly diagnosed by rheumatologists (39.8%) but was diagnosed in 22.3% and 7.3% of cases by general practitioners and dermatologists, respectively (Fig. 6).

## Discussion

In this study, we analyzed occurrences of health events (i.e., diagnosis codes and types of healthcare provider seen by patients) using the MarketScan research databases prior to the diagnosis of PsA to better comprehend a patient’s journey in obtaining a PsA diagnosis. Overall, nonspecific musculoskeletal manifestations and non-PsA diagnoses were common up to 6 years before the diagnosis of PsA compared with controls without PsA. The diagnoses and codes entered prior to PsA diagnosis varied by provider type. Rheumatologists and dermatologists, unsurprisingly, were more likely to code for different types of arthritis and skin manifestations/psoriasis, respectively. Rheumatologists accounted for 40% of the PsA diagnoses, whereas general practitioners and dermatologists combined to make 30% of PsA diagnoses, and the remaining 30% of diagnoses were made by physicians in other specialties.

Compared with controls, patients with PsA in our study received a variety of additional non-PsA diagnosis codes, suggestive of PsA symptom build-up, in the 6 years preceding diagnosis. An analysis of administrative data in Canada similarly reported a build-up of musculoskeletal symptoms as early as 5 years prior to the diagnosis of PsA [15]. Patients manifesting these symptoms have well-documented difficulties (“unable to do” or “with much difficulty”) with various daily tasks, including the inability to bend down to pick up items from the floor or to walk on flat ground [16]. These, in combination with other negative psychological, social, and economic impacts, contribute significantly to the overall burden of disease and poor quality of life [17–20].

Timely referral of a patient with potential PsA for rheumatology evaluation is critical to improving outcomes for patients with PsA, and this has been recognized for some time [9, 21, 22]. The ability to identify PsA symptoms early in the disease course, in addition to recognizing specific trends and patterns in a patient’s pursuit for diagnosis, may permit prompt referral to appropriate specialists, leading to an earlier diagnosis of PsA. These health events may serve as potential predictors of a PsA diagnosis with regard to the many diagnoses received and healthcare providers seen. Identifying the subset of the patient population for screening and referral may be additionally useful to avoid overwhelming rheumatology healthcare providers with high volumes of patients with musculoskeletal symptoms.

Another notable finding in this study was an abrupt increase in the number of psoriasis diagnoses observed preceding the diagnosis of PsA. Although more than two thirds of patients with PsA develop dermatologic lesions prior to joint and musculoskeletal manifestations, the symptoms are not often recognized as related to each other [16, 23, 24]. A prior study reported that almost 70% of patients sought medical help from 1 to 2 healthcare providers and 25% saw ≥ 3 providers within 2 years prior to the survey before receiving a diagnosis of psoriasis [25]. Furthermore, we know that inflammation in the joints and entheses of patients with psoriasis often begins with a subclinical phase without clear physical examination findings [26, 27]. Dermatologists are in a prime position to detect PsA early and rapidly refer patients to rheumatology, but we know there is a gap [28, 29]. In fact, in this study, we saw that dermatologists rarely coded for musculoskeletal findings, potentially suggesting that they are not asking about them or that patients are not reporting musculoskeletal symptoms to their dermatologist. Yet, undiagnosed PsA is commonly observed in dermatology offices [30, 31] and has been recognized in almost one third of patients with psoriasis [29, 31–34]. Patients with psoriasis presenting with PsA manifestations, comprising arthritis, enthesitis, dactylitis, nail disease, and spondylitis, should be evaluated for PsA [35].

Collectively, our study may indicate an opportunity for earlier detection of PsA as characterized by nonspecific musculoskeletal manifestations and skin symptoms and suggests that these musculoskeletal manifestations may be undercoded or underrecognized in clinical practice [36]; improved outcomes have been observed among patients who were promptly treated after receiving a diagnosis of PsA [21, 22, 37]. Approaching PsA diagnosis, general practice, rheumatology, and dermatology visits were much more frequent, suggesting the need for patient education in recognizing symptoms of arthritic conditions. A delay in diagnosis due to initial medical consults with general practitioners and other nonrheumatology healthcare providers is well recognized [8, 16, 18]. An integrated, multidisciplinary method is critical for comprehensive medical care for patients with PsA. Rheumatologists and dermatologists are in an ideal position to mediate collaborations with other healthcare providers and patient advocacy and support groups for the design of a patient-centric intervention and treatment strategy [38, 39]. An improvement in skin and joint symptoms was observed in more than half of patients with psoriasis and PsA after treatment modifications facilitated by rheumatologists and dermatologists [38, 40]. However, as the number of rheumatologists is predicted to decline by 30% by the year 2030 [41], more innovative approaches to patient care are needed. Integrating the use of technology, such as software that calculates composite targets based on both physician- and patient-reported outcomes, to determine disease activity status may be implemented [3]. Validated screening instruments may also be used [42–44], along with patient education and referral guidelines for general practitioners [39, 45]. The future development of applications that tabulate risk or likelihood of disease based on patient data may be a useful undertaking. In creating algorithms using electronic medical records or administrative (i.e., insurance) databases to identify patients who may have early PsA, data scientists need to consider that the diagnosis pathways may differ from patient to patient, depending on the sequence of provider specialties consulted by the patient.

A more patient-centric disease management method may be implemented with the use of currently available diagnostic tools, such as electronic applications allowing the upload of patient-reported outcomes prior to doctor visits [3]. This may help increase patient awareness of symptoms, enhance self-management, improve patient-provider communication, and help predict which patients may be “at risk” or have a high likelihood of actually having PsA. For example, the healthcare provider would enter notes and codes from the visit into the same software, and if an insurer were to use these data, they could send a communication or a pop-up notification to the physician caring for the patient to consider PsA as a potential diagnosis based on the set of codes entered.

There are limitations with our study that should be considered when interpreting the findings. As with any analysis of claims-based data, miscoding by healthcare providers may have occurred; however, the inclusion of patients with PsA who had received 2 diagnosis codes, instead of only 1 diagnosis code, may decrease the likelihood of miscoding or misdiagnosis. Additionally, some healthcare services may not be included in claims databases, leading to the underestimation of overall disease and economic burden. ICD-9-CM and ICD-10-CM codes are used for billing and may not reflect an accurate diagnosis, and there was no independent confirmation of patient diagnoses. Lastly, because this is an analysis of US claims data, these findings may not be fully representative of other countries with different healthcare systems. This analysis is informative, regardless, by evaluating the real-world diagnostic experience of a large number of patients.

## Conclusions

In summary, this study aimed to increase understanding of the patient journey to a diagnosis of PsA and underscores the need for prompt and improved diagnostic approaches from a health systems perspective. An overall prevalence of skin and nonspecific musculoskeletal manifestations, in addition to consultations with various types of healthcare providers, may collectively serve as predictors of PsA. Greater awareness of patterns or trends of health events as seen in our study may alert general practitioners and other healthcare providers in primary care settings to suspect a diagnosis of PsA among their patients. From a research standpoint, patients displaying these predictors may be followed to monitor for PsA development; future analyses will examine sequence of health events as predictors of PsA diagnosis. Using such algorithms may also help raise a differential diagnosis that the physician may not have thought of otherwise. As PsA is associated with considerable healthcare costs [46], the number of non-PsA diagnoses, potentially suggestive of misdiagnoses, and healthcare visits across different types of providers suggest the increased utilization of healthcare resources. The clinical and economic burden associated with PsA will most likely impact disease management and medical and pharmacy policy formulation. Increased awareness and understanding of diagnostic barriers may lead to timelier diagnosis, cost savings, and appropriate intervention to improve outcomes.

## Data Availability

All data generated or analyzed during this study are included in this published article.

## Abbreviations

IA: Inflammatory polyarthropathy
ICD-9-CM and ICD-10-CM: International Classification of Diseases, Ninth and Tenth Revision, Clinical Modification
OA: Osteoarthritis
PsA: Psoriatic arthritis
